# Characterization of Members of the *Fusarium incarnatum*–*equiseti* Species Complex from Natural and Cultivated Grasses Intended for Grazing Cattle in Argentina

**DOI:** 10.3390/jof12010026

**Published:** 2025-12-29

**Authors:** María Julia Nichea, Eugenia Cendoya, Vanessa Gimena Zachetti, Luisina Delma Demonte, María Rosa Repetti, Sofia Alejandra Palacios, María Laura Ramirez

**Affiliations:** 1 Instituto de Investigación en Micología y Micotoxicología (IMICO), Consejo Nacional de Investigaciones Científicas y Técnicas-Universidad Nacional de Río Cuarto (CONICET-UNRC), Ruta 36 Km 601, Río Cuarto 5800, Córdoba, Argentina; juli_nichea@yahoo-com.ar (M.J.N.); ecendoya@exa.unrc.edu.ar (E.C.); vzachetti@exa.unrc.edu.ar (V.G.Z.); spalacios@exa.unrc.edu.ar (S.A.P.); 2 Programa de Investigación y Análisis de Residuos y Contaminantes Químicos (PRINARC), Facultad de Ingeniería Química, Universidad Nacional del Litoral, Santa Fe 3000, Santa Fe, Argentina; luisinademonte@gmail.com (L.D.D.); mrepetti@fiq.unl.edu.ar (M.R.R.)

**Keywords:** zearalenone, novel species, FIESC, *Fusarium*, natural grasses, pastures, phylogenetic study

## Abstract

The detection of zeranol in grazing cattle could be explained by the metabolization of the mycotoxin, zearalenone (ZEA), which was proven to be naturally contaminating the grasses harboring the *Fusarium* species. Previous studies have suggested that members of the *Fusarium incarnatum–equiseti* species complex (FIESC) could be responsible for this contamination. Therefore, the objective of this study is to determine the species composition of FIESC isolates isolated from natural and cultivated pastures previously intended for livestock feed in Argentina and to analyze their ability to produce ZEA. Twenty-five *Fusarium* isolates were characterized by a phylogenetic analysis of the translation elongation factor 1α, and their ZEA production was quantified by cultivation in rice and subsequent analysis by UPLC-MS/MS. The phylogenetic analysis revealed a high genetic diversity identifying five isolates as species already described in the FIESC and six linages which could represent putative new phylogenetic species. In addition, 76% of the isolates were able to produce ZEA, even in high quantities. In conclusion, grasses used for grazing cattle in Argentina harbor a high diversity of FIESC species, many of which are potentially new and capable of producing ZEA, confirming their role as a likely source of this mycotoxin contamination in pastures and improving our understanding of mycological risk in livestock production systems.

## 1. Introduction

Livestock activity is one of the main pillars of the Argentine economy, concentrated mainly in the Pampeana region, followed by the Northeast, Patagonia, the Northwest, and Cuyo. These regions have temperate-to-subtropical climates with rain regimes that allow for the development of grasslands, pastures, and greens that represent the nutritional support of livestock. Extensive beef production in Argentina is mostly based on grazing native and cultivated pastures. Native pastures rely on natural, non-cultivated plant species, offering cost savings, high biodiversity, and soil health benefits, but generally with lower yields and nutrient quality. By contrast, cultivated pastures use introduced species for high productivity and improved feed quality but demand ongoing investment, can reduce biodiversity, and have a limited lifespan [1].

Despite the obvious nutritional value of pastures, they can also pose health risks to grazing animals under certain circumstances, such as kikuyu poisoning in cattle [2,3,4] or when the growth of mycotoxigenic fungi leads to mycotoxin build-up in pastures [5,6,7].

Of particular concern to the Argentinean livestock industry is that the mycotoxin, zearalenone (ZEA), is chemically similar to the growth-promoting α-zearalanol (zeranol), which is banned in Argentina and in the European Union (EU). For the past 20 years, zeranol has been detected in bovine urine during routine analysis of beef cattle farms (enrolled as EU exporters) as part of a national residue control plan by the National Service for Health and Food Quality (SENASA), the central governing authority in Argentina. The cattle were raised on those cattle farms through natural grass grazing, without any external inputs. There is evidence of the presence of zeranol in the urine of deer, goats, sheep, cattle, and horses that only received grass-based feeding [8,9,10]. In Australia and New Zealand, the detection of zeranol in cattle could come from the ZEA metabolism, which is a mycotoxin synthesized by fungi belonging to the genus *Fusarium* and is found naturally in pastures intended for bovine feeding [11,12]. Kennedy et al. [13] and Smith and Morris [14] demonstrated that zeranol (α-ZAL) can be formed from *Fusarium* metabolites *in vivo* in bovine rumen. There is, then, a natural source of zeranol in the pastures, so the finding of zeranol in an animal’s urine alone is not sufficient proof that the producer has abused this substance. In 2011 and 2014, we sampled natural grasses belonging to the family Poaceae from two cattle farms in the Chaco Wetlands, where zeranol had been detected in bovine urine. We found that 90% of the samples were contaminated with ZEA at concentrations ranging from 0.7 to 2120 μg/kg d.m. (mean = 84.5 μg/kg). In addition to α-zearlanol and β-zearalenol (derived from ZEA), other metabolites produced by *Fusarium*, such as toxins T-2 and HT-2, beauvericin (BEA), equisetin, aurofusarin (AUF), neosolaniol (NEO), and diacetoxyscirpenol (DAS), were also detected. The mycological study showed that all samples had *Fusarium* contamination (60–100%), with the most frequently found species being a novel one within the *Fusarium sambucinum* species complex (FSASC), *F. chaquense*. This species is characterized by producing T-2 toxin, T-2 triol, T-2 tetraol, toxin HT-2, DAS, NEO, AUF, and BEA, but not ZEA [15]. The second most frequent isolated species belongs to the *Fusarium incarnatum–equiseti* species complex (FIESC), and we hypothesized that members of this complex could be responsible for the ZEA present in the grasses.

In recently published systematic reviews on the worldwide occurrence levels of mycotoxins in pastures (cultivated and natural), the data presented indicate that mycotoxins produced by the genus *Fusarium* were the most frequent ones, being reported in all articles evaluated. ZEA was the most prevalent mycotoxin, followed by trichothecenes [16,17].

We have also carried out other studies in natural and cultivated grasses intended for grazing cattle, searching for *Fusarium* endophytes [18]. These surveys have revealed that the most commonly isolated species were included in the FIESC. However, some strains in the complex could not be satisfactorily identified to the species level using morphological markers. In order to identify them, molecular biological techniques were used through phylogenetic analysis. Thus, those species are known as “phylospecies”.

Multilocus phylogenetic analyses revealed that the FIESC had more than 40 phylogenetically distinct species which had been divided into the *Equiseti* clade and the *Incarnatum* clade [19,20]. They are distributed throughout tropical, subtropical, and temperate regions. Several studies have revealed that members of the FIESC are capable of producing mycotoxins. These fungi can synthesize a range of toxic secondary metabolites, such as nivalenol (NIV) and its acetylated derivative (4-acetyl- nivalenol; 4-ANIV), deoxynivalenol (DON) and its acetylated derivatives (15-acetyldeoxynivalenol and 3-acetyldeoxynivalenol, 15-ADON and 3-ADON), DAS, NEO, ZEA, BEA, and other emerging toxins [21]. Therefore, the aims of this work were to understand the species composition of the endophyte FIESC isolates previously obtained from natural and cultivated grasses used for cattle production in Argentina and to analyze the capability of these isolates to produce ZEA.

## 2. Materials and Methods

### 2.1. Origin of Isolates Examined

Twenty-five *Fusarium* isolates were recovered from the culture collection of the Laboratory of Mycology, Department of Microbiology and Immunology, Universidad Nacional de Rio Cuarto (RC). The isolates were previously isolated as endophytes from the aerial part of natural and cultivated asymptomatic grasses (Poaceae) devoted to cattle grazing obtained from beef cattle farms in Argentina. In the particular case of natural grasses, the selection criteria used was to sample the most palatable ones (Table 1). All of these isolates were putatively assigned to the FIESC based on their similarity to the colony color and micromorphological characteristics described by Leslie and Summerell [22] for *F. equiseti* and *F. semitectum*, which represent the diagnostic morphotypes for members of this species complex.

### 2.2. DNA Extraction, Amplification and Sequencing

Total genomic DNA was extracted using the cetyltrimethylammonium bromide (CTAB; Sigma-Aldrich, St. Louis, MO, USA) method, as described by Leslie and Summerell [22]. In brief, all the isolates were grown in 50 mL of complete medium (CM) and incubated in an orbital shaker (150 rpm) for 3 d at 25 °C. The resulting mycelia were harvested by filtration through non-gauze milk filters (KenAG, Ashland, OH, USA). Excess water was removed by blotting the mycelia between clean paper towels, and the dried mycelia were stored frozen at −20 °C until ground and extracted with CTAB. The quality of the genomic DNA was determined by electrophoresis and quantified using a spectrophotometer (model ND-1000; NanoDrop Technologies, Wilmington, DE, USA).

Partial amplification of the translation elongation factor 1-α (*TEF1*) gene was carried out with PCR primers EF1 and EF2 using the amplification conditions described by O’Donnell et al. [23]. The PCR products were purified and sequenced by Macrogen, Inc. (Seoul, Republic of Korea), using the same primers used for the PCR amplification. Sequences were edited using the BioEdit Sequence Alignment Editor 7.1.3.0 [24]. Initial identifications of all *Fusarium* isolates relied on BLAST (https://blast.ncbi.nlm.nih.gov, accessed on 30 July 2025) search comparisons against the Fusarium MLST database (https://fusarium.mycobank.org, accessed on 5 August 2025). These results were then used to produce a reference dataset for the FIESC, using previously deposited sequences obtained from the NCBI nucleotide database. All sequences generated in this study were deposited in GenBank, with the accession numbers provided in Table 1.

### 2.3. Sequence Analysis and Phylogenetic Analysis

Multiple sequence alignment of the *TEF1* gene was performed using the Web-based program MAFFT (https://mafft.cbrc.jp/alignment/server/, accessed on 5 August 2025) [25]. *TEF1* sequences from the reference FFSC strains and other *Fusarium* species obtained from GenBank were included in the analysis (Table 2). Based on this alignment, phylogenetic analyses were performed to selected strains by maximum likelihood (ML), using PhyML 3.1 [26], and Bayesian inference (BI), using MrBayes 3.2.6 [27]. For ML and BI analyses, the best substitution model was determined using jModelTest 2.1.10 [28] and scored following the Akaike information criterion (AIC). The TrN + G model was used. For the ML analysis, the robustness of the best tree was evaluated by 1000 bootstrap replications. For the BI analysis, two runs with four chains each were run for 10 million generations with a sampling frequency of every 100 generations. Trees after the initial 25% of trees for each run were discarded as burn-in. *Fusarium longipes* NRRL 20695 was used as outgroup.

### 2.4. Zearalenone Production

All the isolates were cultured in Erlenmeyer flasks (250 mL) containing 25 g of long grain rice. Ten ml of distilled water was added before autoclaving for 30 min at 121 °C in order to reach 40% humidity. The procedure was repeated twice. Each flask was inoculated with a 3-mm diameter agar disk taken from the margin of a colony grown on synthetic nutrient agar (SNA) at 25 °C for 7 days [22]. Flasks were shaken by hand once a day for 1 week. These cultures were incubated for 28 days at 25 °C in dark. Non-inoculated flasks with rice were used as a negative control. At the end of the incubation period, the contents of the flask were dried at 50 °C for 24 h, ground to a fine powder, and then stored at −20 °C until analyzed for mycotoxins.

### 2.5. Zearalenone Analysis

QuEChERS extraction procedure was followed according to Lacina et al. [29] with the modifications made by Dzuman et al. [30]. The methodology was applied as follows: a 3 g portion of the homogenized sample was weighed in a polypropylene centrifugation tube and acidified Milli-Q water (10 mL, 0.2% formic acid) was added and left to soak the matrix for 30 min. Then, acetonitrile was added (10 mL), and the sample was extracted for 30 min using a laboratory shaker (New Brunswick Scientific, Edison, NJ, USA). Next, 4 g of magnesium sulfate and 1 g of sodium chloride were added and the tube was shaken for 1 min, followed by centrifugation (5 min, 10,000 rpm; Thermo Fisher Scientific Inc., Waltham, MA, USA). The organic upper layer (2 mL) was removed and shaken with 0.1 g of Bondesil-C_18_ and 0.3 g of magnesium sulfate for 2 min, followed by centrifugation (5 min, 10,000 rpm). Finally, 1 mL of purified extract was removed into a vial prior to injection on an LC–MS system.

The detection and quantitation of ZEA was performed using an ACQUITY UPLCTM system (Waters Corporation, Milford, MA, USA) coupled with a triple quadrupole mass spectrometer (MS/MS) equipped with a Z-spray electrospray ionization source (ESI). Chromatographic separation was achieved on a BEH C18 reversed-phase column (1.7 μm, 2.1 × 100 mm) maintained at 40 °C, utilizing a gradient elution with mobile phases comprising ultrapure water with 5 mM NH_4_F and 0.1% formic acid (A) and methanol with similar additives (B). The flow rate was set to 0.4 mL/min, and the injection volume was 10 μL. The MS/MS detection was carried out in positive mode, monitoring transitions *m*/*z* 319.1 → 187.0 (quantifier) and 319.1 → 185.0 (qualifier), with collision energies optimized accordingly. Quantification was performed using matrix-matched calibration curves over a concentration range from 1 to 300 ng/mL, following validation parameters including recovery, repeatability, linearity (R^2^ > 0.99) (Figure S1), and limits of detection and quantification at signal-to-noise ratios of 3 and 10, respectively. The method demonstrated the limits of detection and quantification of 3.0 ng/g and 15 ng/g, respectively, and showed high precision, with mean recoveries ranging from 85% to 98%. The entire procedure ensures the accurate, sensitive, and reproducible determination of ZEA in the analyzed samples.

## 3. Results

### 3.1. Phylogenetic Analyses

**Figure 1:**
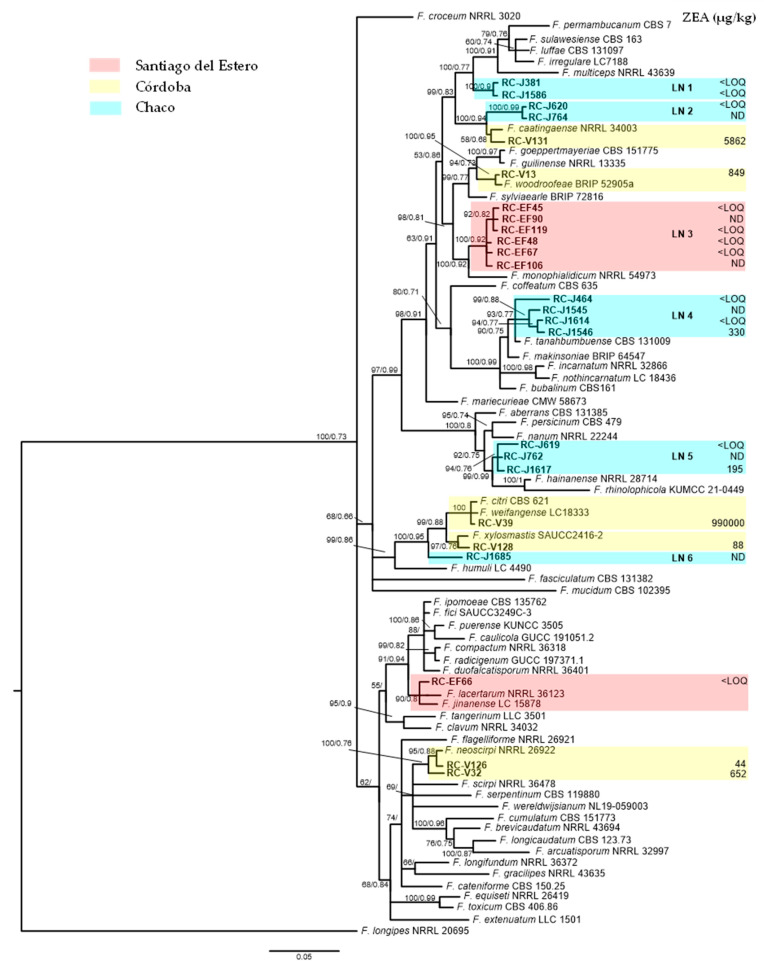
Bayesian phylogenetic tree inferred from partial *TEF1* sequences showing the phylogenetic relatedness of *Fusarium* species isolated from Poaceae plants with other species of the *Fusarium incarnatum–equiseti* species complex. Bayesian posterior probability scores ≥ 0.95, followed by ML bootstrap values ≥ 0.70 are shown at the internodes. *F. longipes* NRRL 20695 was used as the outgroup. Zearalenone production (µg/kg) of *Fusarium* isolates is indicated next to each isolate. ND not detected (<LOD), (<LOQ: 15 ng/g). The origin of the isolates is indicated in color.

A phylogenetic analysis based on the *TEF1* gene showed that the 25 isolates belonged to the FIESC (Figure 1). Part of the isolates (seven) were identified as the *F. caatingaense* (RC-V131), *F. woodroofeae* (RC-V13), *F. weifangense* (RC-V39), *F. xylosmatis* (RC-V128), *F. neoscirpi* (RC-V126 and RC-V32), and *F. lacertarum* (RC-EF66) phylospecies. Interestingly, we also found that most of the isolates could not be resolved within any phylospecies and form five monophyletic groups, provisionally called LN1 to LN5. The group LN1 (two isolates) clustered closer to the phylospecies *F. multiceps*, and LN2 (two isolates) shared a clade with the sister species *F. caatingaense*. The largest group, LN3 (six isolates), clustered closer to *F. monophialidicum*; LN4 (four isolates) was closely related to *F. tanahbumbuense*. While LN5 (three isolates) was closely related to *F. hainanense*; one isolate, RC-J1617, formed a singleton lineage (LN6) (Figure 1).

### 3.2. ZEA Production

In general, 76% (19/25) of the endophyte isolates belonging to the FIESC isolated from natural and cultivated grasses were able to produce ZEA. The highest level was shown by the *F. weifangense* isolate RC-V39 (990,000 µg/kg) (Figure S2). Other isolates that also produce large amounts of ZEA included *F. caatingaense* RC-V131 (5862 µg/kg), *F. woodroofeae* RC-V13 (849 µg/kg) (Figure S3), both *F. neoscirpi* RC-V32 and RC-V126, 652 µg/kg and 44 µg/kg, respectively (Figure S4), and *F. xylosmatis* RC-V128 (88 µg/kg). Most of the remaining isolates included in the novel lineages were able to produce low levels of ZEA (<LOQ > LOD), except for two isolates, RC-J1546 (LN4), which produced 330 µg/kg, and RC-J1617 (LN5), which produced 195 µg/kg (Figure 1).

## 4. Discussion

This is the first study to elucidate the phylogeny and the potential of ZEA production for 25 endophyte isolates belonging to the FIESC obtained from natural and cultivated grasses intended for grazing cattle in Argentina. The phylogeny suggested the occurrence of five known species in the FIESC (*F. caatingaense*, *F. woodroofeae*, *F. weifangense*, *F. xylosmatis*, *F. neoscirpi*, and *F. lacertarum*) and six lineages (one singleton, the rest with several isolates) representing putatively novel phylogenetic species. Finding several *Fusarium* species that may represent new taxa requires further confirmation using multilocus phylogenetic analyses combined with morphology. The genetic diversity found in our survey of the FIESC isolates from natural and cultivated grasses was high and confirmed the previous reports on the FIESC that showed high biological variability existing within this species complex isolated from multiple hosts. Members of the FIESC have been frequently associated with native and wild grasses [31,32,33,34,35,36,37] and commonly isolated from wheat, maize, rice, soybean, barley, and oat [38,39,40,41,42]. FIESC species can produce a dozen mycotoxins, mainly trichothecenes and ZEA [21,38,43].

It was noticeable that each novel lineage contained isolates obtained in the same geographical area. For example, LN3 has six members, all isolated from cultivated grass, *Megathyrsus maximus* (syn. *Panicum maximum*), from Santiago del Estero province. Additionally, the results showed that natural grasses (isolates from Córdoba and Chaco provinces) exhibit a greater diversity compared to the cultivated grass *M. maximus*.

In the case of the isolates from natural grasses in the Chaco province, all of them were clustered in five novel lineages (one singleton). These isolates were obtained in a previous study that we conducted in the east of Chaco Province in Argentina, in a wetland ecosystem formed by the Paraná and Paraguay river floodplain that is one of the three most biodiverse biomes in the country. As part of this study, we sampled asymptomatic Poaceae plants (*n* = 175) representative of 12 grass genera. All the grasses were naturally contaminated with *Fusarium* mycotoxins, including, mainly, ZEA and the type A trichothecenes, such as T-2 and HT-2 [12]. All the metabolites noted above are reported to be produced by various *Fusarium* species [21]. The mycological analysis revealed that 60–100% of the sampled plants were contaminated with *Fusarium*, the most prevalent one being a novel species, *F. chaquense*, that belongs to FSAMSC. This species was responsible for type A trichothecene grass contamination, since all the isolates were able to produce *in vitro* this kind of mycotoxin, and we were also able to demonstrate the presence of the biosynthetic genes [15]. The second most frequently isolated species belonged to the FIESC, which is part of the present study. We have demonstrated that 67% of these isolates (8/12) were capable of in vitro ZEA production, and we believe that they are responsible for grass contamination with this mycotoxin.

It is noteworthy that three of the known species found, such as *F. weifangense*, *F. xylosmatis*, and *F. woodroofeae*, have recently been described, and almost all the recorded strains are from China [20,42,44]. In particular, *F. weifangense* was first isolated from symptomatic tissues of *Triticum aestivum* [40], has recently been reported from necrotic spots of *Prunus salicina* [20], and causes fruit rot diseases in the Indian jujube in Thailand [45], always as a pathogen, never as a grass endophyte. *Fusarium xylosmatis*, which has also been recently described from necrotic spots of *Xylosma congesta* in China, is closely related to *F. weifangense* [20]. Neither of these three species has been reported as ZEA producers before. During the present study, all the isolates belonging to these species were able to produce high levels of ZEA, with *F. weifangense* RC-V39 being the highest producer (990,000 µg/kg).

*Megathyrsus maximus* is a Poaceae plant originally from Africa and is widely used as forage grass in Brazil and Argentina. There is only one previous report on endophytic *Fusarium* on *M. maximum* seeds collected in several geographic locations in Brazil. The two species found were included in the FIESC: *F. hainanense* and *F. duofalcatisporum* [34]. In the present study, we did not obtain these species; most of the *Fusarium* endophytes isolated from *M. maximus* clustered in the LN3, and one was identified as *F. lacertarum*. This species has previously been associated with *Fusarium* head blight on sorghum in the USA [46] and causing wilt in *Vigna unguiculata* in Brazil [47], and has also been reported in this country as a pathogen in *Nopalea cochenillifera* [48]. However, mycotoxin characterization in *F. lacertarum* has not been performed. Our strain (RC-EF66) was able to produce ZEA at very low concentrations.

We observed one isolate (RC-V13) that clustered with the known FIESC phylospecies, *F. caatingaense*, formally described in 2019 in Brazil, associated with *Dactylopius opuntiae* (Hemiptera: Dactylopiidae) [49], and also reported in cultivated rice [50]. *F. caatingaense* strains from rice and insects have been reported as producers of BEA, FUS, AcDON, DON, T-2, DAS, NIV, and ZEA [39,50]. In the present study, the *F. caatingaense* strain was also capable of producing ZEA at a high level.

Two isolates clustered with *F. neoscirpi* (RC-V32 and RC-V126), and both isolates were capable of ZEA production. This species was formally described by Xia et al. [51] as a unique single strain from soil.

## 5. Conclusions

The results of the present study enhance our understanding of FIESC presence as an endophyte in natural and cultivated grasses used for grazing cattle and also the ZEA production by the different species found. It is noticeable in the presence of six putative novel lineages. This survey shows that naturally occurring and cultivated grasses not only harbor a high diversity of known species within the FIESC, which are pathogens of rice, wheat, and other hosts, but novel *Fusarium* species, which have the capability to produce mycotoxins.

## Figures and Tables

**Table 1:** *Fusarium incarnatum–equiseti* species complex isolates obtained from grasses used in the current study.

Strain Code	Year	Host	Geographic Origin	GPS Coordinates	GenBank Accession Number *TEF1*
RC-J381	2011	*Poaceae*	Chaco	27°31′36.8″ S, 59°05′03.8″ W	PX363448
RC-J464	2011	*Poaceae*	Chaco	27°31′25.6″ S, 59°05′00.5″ W	PX363449
RC-J762	2011	*Poaceae*	Chaco	27°33′54.4″ S, 60°24′44.6″ W	PX363452
RC-J619	2011	*Poaceae*	Chaco	27°33′28.2″ S, 60°25′05.3″ W	PX363450
RC-J620	2011	*Poaceae*	Chaco	27°33′28.2″ S, 60°25′05.3″ W	PX363451
RC-J764	2011	*Poaceae*	Chaco	27°33′54.4″ S, 60°24′44.6″ W	PX363453
RC-J1586	2014	*Spartina* sp.	Chaco	27°30′49.5″ S, 59°05′06.1″ W	PX363454
RC-J1545	2014	*Chloris* sp.	Chaco	27°30′49.5″ S, 59°05′06.1″ W	PX363458
RC-J1546	2014	*Chloris* sp.	Chaco	27°30′49.5″ S, 59°05′06.1″ W	PX363459
RC-J1685	2014	*Eragrostis* sp.	Chaco	27°31′07.0″ S, 59°05′00.9″ W	PX363457
RC-J1614	2014	*Panicum* sp.	Chaco	27°30′49.5″ S, 59°05′06.1″ W	PX363455
RC-J1617	2014	*Panicum* sp.	Chaco	27°30′49.5″ S, 59°05′06.1″ W	PX363456
RC-EF45	2022	*Megathyrsus maximus*	Santiago del Estero	27°08′02.07″ S, 61°51′34.94″ W	PX363441
RC-EF90	2022	*Megathyrsus maximus*	Santiago del Estero	27°08′02.07″ S, 61°51′34.94″ W	PX363445
RC-EF119	2022	*Megathyrsus maximus*	Santiago del Estero	27°08′02.07″ S 61°51′34.94″ W	PX363447
RC-EF48	2022	*Megathyrsus maximus*	Santiago del Estero	27°08′02.07″ S, 61°51′34.94″ W	PX363442
RC-EF67	2022	*Megathyrsus maximus*	Santiago del Estero	27°08′02.07″ S, 61°51′34.94″ W	PX363444
RC-EF106	2022	*Megathyrsus maximus*	Santiago del Estero	27°08′02.07″ S, 61°51′34.94″ W	PX363446
RC-EF66	2022	*Megathyrsus maximus*	Santiago del Estero	27°08′02.07″ S, 61°51′34.94″ W	PX363443
RC-V13	2016	*Aristida laevis*	Córdoba	32°44.661′ S, 64°47.251′ W	PX363463
RC-V32	2016	*Paspalum quadrifarium*	Córdoba	32°44.805′ S, 64°46.440′ W	PX363464
RC-V39	2016	*Paspalum notatum*	Córdoba	32°44.803′ S, 64°46.440′ W	PX363462
RC-V126	2016	*Melinis repens*	Córdoba	32°44.806′ S, 64°46.697′ W	PX363460
RC-V128	2016	*Panicum* sp.	Córdoba	32°44.727′ S, 64°46.706′ W	PX363465
RC-V131	2016	*Aristida laevis*	Córdoba	32°44.661′ S, 64°47.251′ W	PX363461

**Table 2:** Source information for *TEF1* reference sequences used in the phylogenetic analyses.

*Fusarium* Species	Strain Number	GenBank Accession Number *TEF1*
*F. aberrans*	CBS 131385	MN170445.1
*F. arcuatisporum*	NRRL 32997	GQ505624
*F. brevicaudatum*	NRRL 43694	GQ505665
*F. bubalinum*	CBS 161.25	MN170448
*F. caatingaense*	NRRL 34003	MN170449.1
*F. cateniforme*	CBS 150.25	MN170451
*F. caulicola*	GUCC 191051.2	OR043884.1
*F. citri*	CBS 621.87	MN170452.1
*F. clavum*	NRRL 34032	GQ505635
*F. coffeatum*	CBS 635.76	MN120755.1
*F. compactum*	NRRL 36318	GQ505646
*F. concolor*	NRRL 13459	GQ505674.1
*F. croceum*	NRRL 3020	GQ505586
*F. cumulatum*	CBS 151773	OR671087.1
*F. duofalcatisporum*	NRRL 36401	GQ505651
*F. equiseti*	NRRL 26419	GQ505599.1
*F. extenuatum*	LLC1501	OP487158.1
*F. fasciculatum*	CBS 131382	MN170473.1
*F. fici*	SAUCC3249C-3	PQ309133.1
*F. flagelliforme*	NRRL 26921	GQ505600
*F. goeppertmayeriae*	CBS 151775	OR670981.1
*F. gracilipes*	NRRL 43635	GQ505662.1
*F. guilinense*	NRRL 13335	GQ505590
*F. hainanense*	NRRL 28714	MN170510.1
*F. humuli*	LC4490	MK289614.1
*F. incarnatum*	NRRL 32866	GQ505615
*F. ipomoeae*	CBS 135762	MN170478.1
*F. irregulare*	LC7188	MK289629.1
*F. jinanense*	LC15878	OQ125131.1
*F. lacertarum*	NRRL 36123	GQ505643
*F. longicaudatum*	CBS 123.73	MN170481
*F. longifundum*	NRRL 36372	GQ505649
*F. longipes*	NRRL 20695	GQ915509
*F. luffae*	CBS 131097	MN170482.1
*F. makinsoniae*	BRIP 64547	OQ626867
*F. mariecurieae*	CMW 58673	OR671063.1
*F. monophialidicum*	NRRL 54973	MN170483
*F. mucidum*	CBS 102395	MN170485.1
*F. multiceps*	NRRL 43639	GQ505666.1
*F. nanum*	NRRL 22244	MN170486.1
*F. neoscirpi*	NRRL 26922	GQ505601
*F. nothincarnatum*	LC18436	OQ125147
*F. pernambucanum*	CBS 791.70	MN170491.1
*F. persicinum*	CBS 479.83	MN170495.1
*F. puerense*	KUNCC 3505	PV464279.1
*F. radicigenum*	GUCC 197371.1	OR043907.1
*F. rhinolophicola*	KUMCC 21-0449	OR026001.1
*F. scirpi*	NRRL 36478	GQ505654
*F. serpentinum*	CBS 119880	MN170499
*F. sulawesiense*	CBS 163.57	MN170501.1
*F. sylviaearle*	BRIP 72816	OR269444
*F. tanahbumbuense*	CBS 131009	MN170506.1
*F. tangerinum*	LLC3501	OP487189.1
*F. toxicum*	CBS 406.86	MN170508
*F. weifangense*	LC18333	OQ125107.1
*F. wereldwijsianum*	NL19-059003	MZ921852.1
*F. xylosmatis*	SAUCC2416-2	PQ309132.1

## Data Availability

All sequences generated in this study were deposited in GenBank, with the accession numbers provided in Table 1. The original contributions presented in this study are included in the article and Supplementary Materials. Further inquiries can be directed to the corresponding author.

## Supplementary Materials

The following supporting information can be downloaded at: https://www.mdpi.com/article/10.3390/jof12010026/s1, Figure S1: Matrix-matched calibration (red) and standard solution calibration (blue) curves for zearalenone (ZEA); Figure S2: *Fusarium weifangense* RC-V32. Quantifier transition (top) and qualifier transition (botton). q/Q = 1.46; Figure S3: *Fusarium woodroofeae* RC-V13. Quantifier transition (top) and qualifier transition (botton). q/Q = 1.36; Figure S4: *Fusarium neoscirpi* RC-V32 (a) and RC-V126 (b) Quantifier transition (top) and qualifier transition (botton). q/Q = 1.77.
