# Supplementary material for: Characterization of Members of the Fusarium incarnatum–equiseti Species Complex from Natural and Cultivated Grasses Intended for Grazing Cattle in Argentina
Source: J Fungi (Basel). 2025 Dec 29;12(1):26. doi: 10.3390/jof12010026 (PMC12843072; doi:10.3390/jof12010026)
Supplement: Supplementary file 1 [file jof-12-00026-s001.zip › jof-4062005-supplementary.pdf]

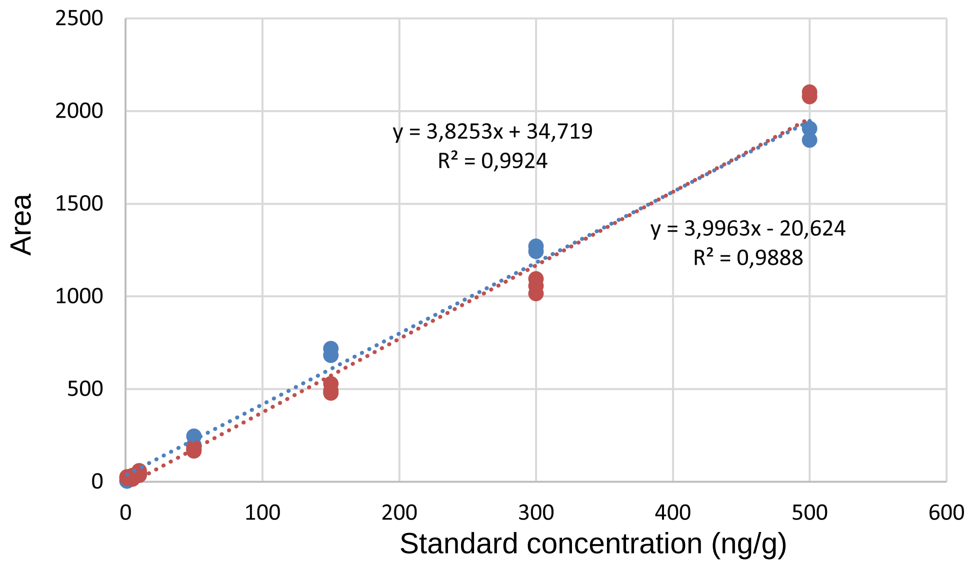

Figure S1: Matrix-matched calibration (red) and standard solution calibration (blue) curves for zearalenone (ZEA)

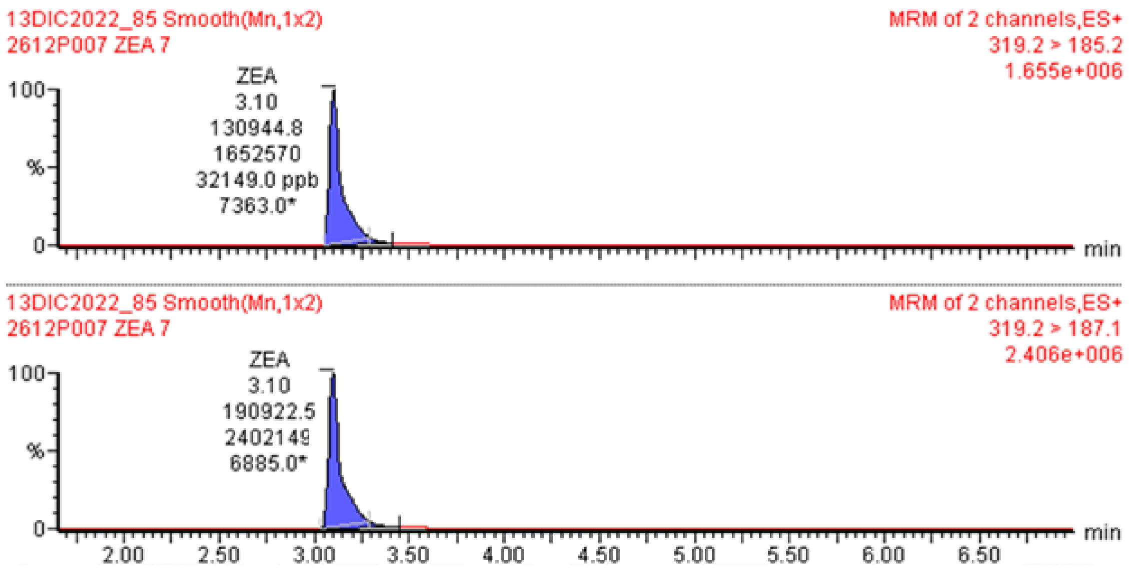

Figure S2. *Fusarium weifangense* RC-V32. Quantifier transition (top) and qualifier transition (botton). q/Q = 1.46

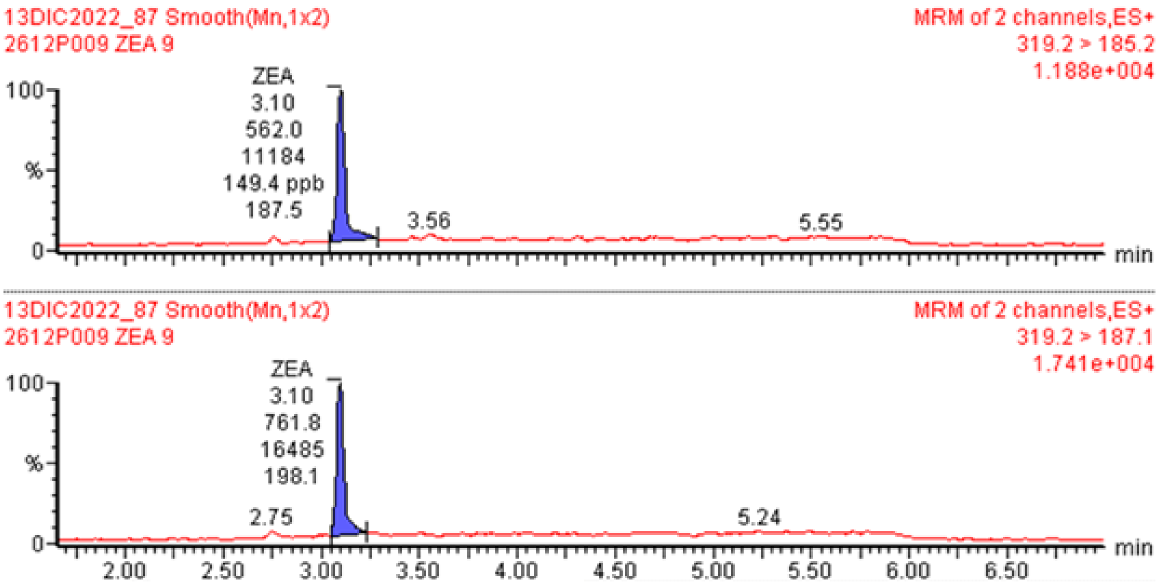

Figure S3. *Fusarium woodroofeae* RC-V13. Quantifier transition (top) and qualifier transition (botton). q/Q = 1.36.

a

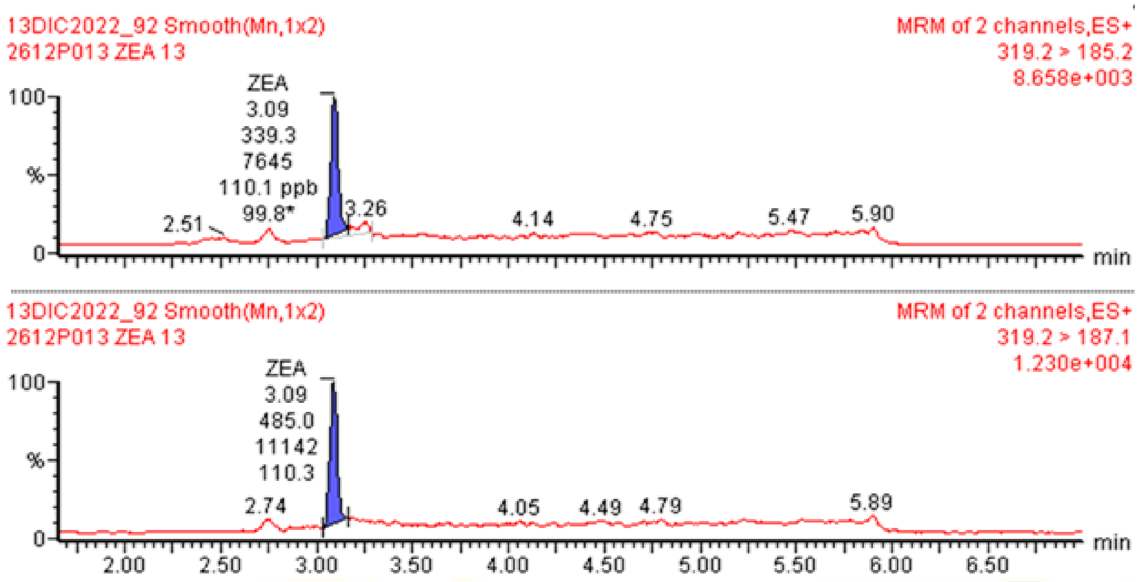

I

b

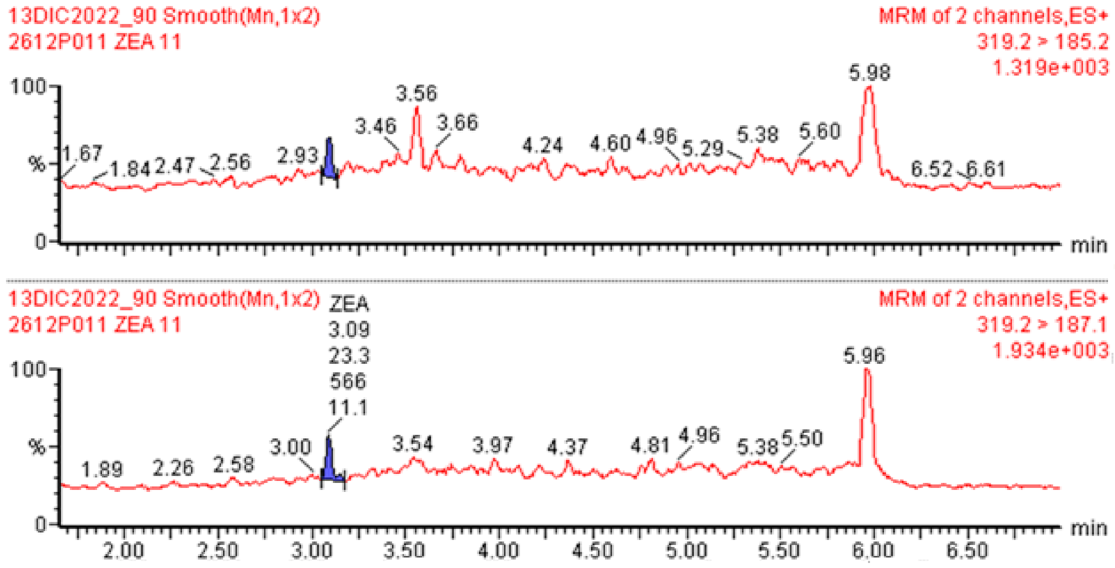

Figure S4.: *Fusarium neoscirpi* RC-V32 (a) and RC-V126 (b) Quantifier transition (top) and qualifier transition (botton). q/Q = 1.77
